# Impact of thermodynamical rotational flow of cerebrospinal fluid in the presence of elasticity

**DOI:** 10.1186/s13104-023-06602-w

**Published:** 2023-11-29

**Authors:** Hemalatha Balasundaram

**Affiliations:** Department of Mathematics, Rajalakshmi Institute of Technology, Chembarambakkam, Chennai, Tamil Nadu 600124 India

**Keywords:** Brain parenchyma, Cerebrospinal fluid, Elasticity, Hydrocephalus, Solute reaction, Soret effect, Rotational flow

## Abstract

**Objective:**

To explore the experimental justification of cerebrospinal fluid (CSF) amplitude and elastic fluctuations of ventricles, we extend our previous computational study to models with rotational flow and suitable boundary conditions. In the present study, we include an elastic effect due to the interaction with the thermal solutal model which accounts for CSF motion which flows rotationally due to hydrocephalus flows within the spinal canal.

**Methods:**

Using an analytical pertubation method, we have attempted a new model to justify CSF flow movement using the influences of wall temperature difference.

**Results:**

This paper presents results from a computational study of the biomechanics of hydrocephalus, with special emphasis on a reassessment of the parenchymal elastic module. CSF amplitude in hydrocephalus patients is 2.7 times greater than that of normal subjects.

**Conclusions:**

This finding suggests a non-linear mechanical system to present the hydrocephalic condition using a numerical model. The results can be useful to relieve the complexities in the mechanism of hydrocephalus and can shed light to support clinically for a convincing simulation.

**Supplementary Information:**

The online version contains supplementary material available at 10.1186/s13104-023-06602-w.

## Introduction

Hydrocephalus is the imbalance between the production and absorption of cerebrospinal fluid (CSF). The prevalence of hydrocephalus is 0.3% to 0.5% for every 1000 patients per year [1]. There are many complexities in the brain pathology and mechanism of hydrocephalus. Due to the limitations of in vivo studies, it is not possible to study this disorder in various clinical conditions since it leads to damage to their health condition. it is not possible to take accurate undistorted measurements while using a clinical method. Therefore, reducing the complex mathematical modeling becomes an important method for studying CSF biofluid with various parameters. The simulation of the temperature effects on hydrocephalus CSF in oscillating flow that affects high pressure is of importance. We tried to prove these using previous studies to simulate the hydrocephalic condition using computational fluid dynamic and fluid-structure interaction methods [2–26]. However, some of them calculate the effect of temperature in their simulations [3, 27–33].

Previous thermal analyses of hydrocephalus patients have not considered the effect of rotational flow and elasticity of the ventricular walls. Recently, we have studied the CSF flow with elastic boundary moving in a laminar path bounded by a porous layer for hydrocephalus patients. In the present study, we include an elastic effect due to the interaction with the thermal solutal model which accounts for CSF motion which flows rotationally due to hydrocephalus flows within the spinal canal. Using an analytical method, we have attempted a new model to justify CSF flow movement using the influences of wall temperature difference. We used hydroceplaus patients refered in [4, 17, 33] for this simulation.

## Mathematical formulation

**Fig. 1:**
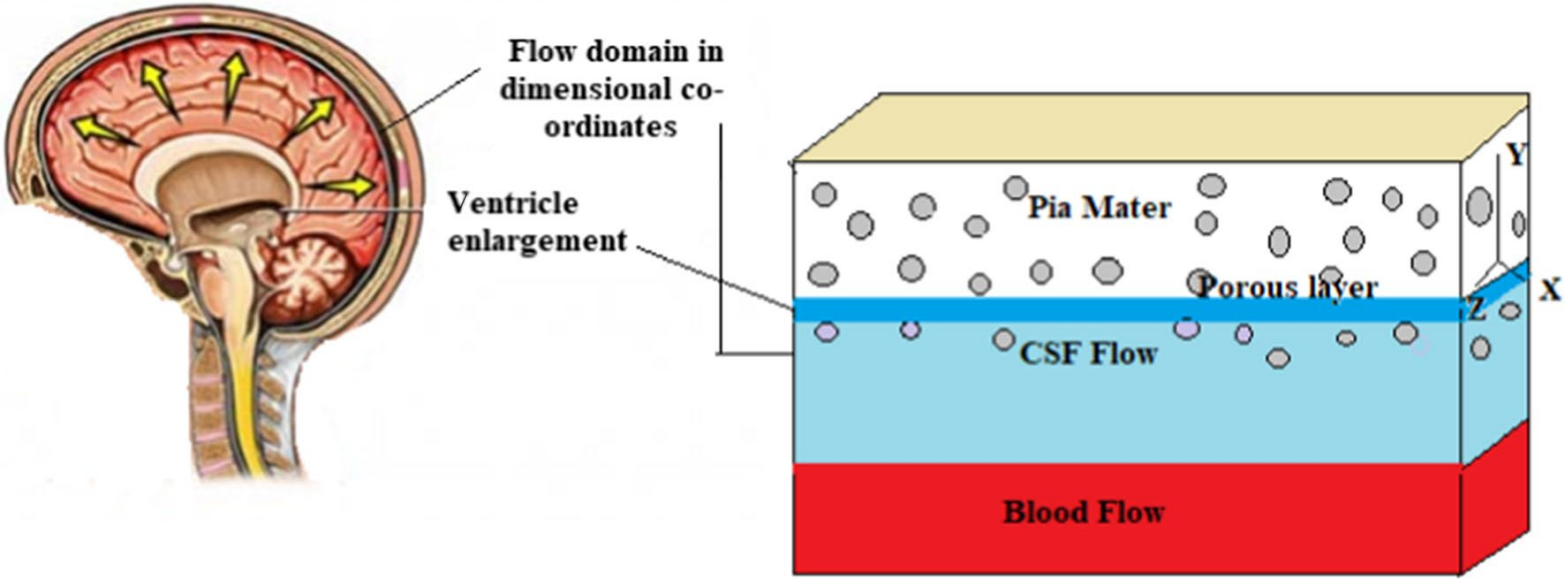
Schematic representation of CSF flow in the ventricular region

Due to the geometric complexity of the flow in the cranial system, many of them attempt to show CSF flow and have analyzed the system with multiple compartments, developed as a simple geometry as a cylindrical tube. Considering these compartments in our mind to qualify the physical configuration Fig. 1 is a closed cylindrical model through which the flow has been developed. In this approach, governing equations are solved analytically [2]. We consider the CSF oscillating flow passes through porous parenchyma. We take $z$ as the vertical axis and $y$ chosen as perpendicular to it.

We consider the hydrodynamic incompressible, unsteady viscous fluid moving vertically in a finite medium embedded by brain parenchyma. Let $\partial {\Omega }_{b}$ represents the boundary of ${\Omega }_{b}$ which represents the CSF flow surface. Also, we describe the coordinate system in such a way that the horizontal and vertical directions on the *y-axis* and *z-axis* respectively. Fluid particle rotating in a uniform velocity $\Omega$ with respect to *y-axis*. Consequently, $u$ and $w$ are denoted as the velocity of fluid flow in $y$ and $z$ respectively. Considering the fluid initially at rest portion with uniform temperature and concentration and all the physical quantities depend only on $y$ and $t$. Then the fluid particle starts moving at a time $t$ and it is maintained uniformly throughout the fluid moment. Without loss of generality, we neglect pressure gradient and gravitational forces in the fluid flow for our convenience.

The physical principles of rigorous CSF fluid flow physics are used to develop the flow and thermal diffusivity defined in the analytical construction from the previous research papers. we tried to show the acceleration of the Soret parameter as there is an increase in the temperature of fluid flow. Hence this conservation stabilizes that lead to a system of non-linear partial differential equations (PDE) as the conservation of mass and Navier Stokes with heat and diffusivity equation are given by

Equation of Mass [2]

1 $$\frac{\partial u}{\partial y}+\frac{\partial w}{\partial z}=0$$

Equation of motion [11] with rotational effect

2 $$\frac{\partial u}{\partial t}+G{w}_{0}\frac{\partial u}{\partial y}+2{{\Omega^\prime }}u=-\frac{1}{\rho }\frac{\partial p}{\partial x}+\nu \left(\frac{{\partial }^{2}u}{{\partial y}^{2}}\right) -\frac{\nu }{K} u-\frac{RN}{\rho }u$$

3 $$\frac{\partial w}{\partial t}+{Gw}_{0}\frac{\partial w}{\partial y}-2{{\Omega^\prime }}w=-\frac{1}{\rho }\frac{\partial p}{\partial x}+\nu \left(\frac{{\partial }^{2}w}{{\partial y}^{2}}\right)-\frac{\nu }{K} w -\frac{RN}{\rho }w$$

Energy Eq. [28] with a heat conduction parameter

4 $$\left(\frac{\partial T}{\partial t}+{w}_{0}\frac{\partial T}{\partial y}\right) =\frac{k}{\rho {C}_{p}}\left(\frac{{\partial }^{2}T}{{\partial y}^{2}}\right)+\frac{J}{\rho {C}_{p}}(T-{T}_{c})$$

Concentration Eq. [16] with Soret effect

5 $$\frac{\partial C}{\partial t}+{w}_{0}\frac{\partial C}{\partial y}=D\frac{{\partial }^{2}C}{{\partial y}^{2}}+\frac{D {K}_{T}}{{T}_{m}}\left(\frac{{\partial }^{2}T}{{\partial y}^{2}}\right)$$

6 $$u=0,w=0,T={T}_{0},C={C}_{0}\mathrm \ at \,y\,=\,0 \,and\, t\le 0$$

$$u={u}_{0}\left(1 + cos\lambda t\right), w=0,T={T}_{c},C={C}_{c} \,at \,y\,=\,1 ,t\,>\,0$$

Non-dimensional quantities relevant to the problem and neglecting the dash symbol for our convenience

$${y}{\prime}=\frac{y}{l}, {t^\prime}=\frac{t{w}_{0}}{l}, {w^\prime}=\frac{wl}{{w}_{0}}, {u^\prime}=\frac{u}{{u}_{0}}$$

$${\theta^\prime}=\frac{T-{T}_{c}}{{T}_{0}-{T}_{c}}, {\phi^\prime}=\frac{C-{C}_{c}}{{C}_{0}-{C}_{c}}, Sc=\frac{\nu }{D}, Re=\frac{{w}_{0}l}{\nu }, Sr=\frac{D {K}_{T}}{{T}_{m}\nu }\left(\frac{{T}_{0}-{T}_{m}}{{C}_{0}-{C}_{m}}\right)$$

$${G}_{pm}=\frac{RN{l}^{2}}{\mu }, Pe=\frac{\rho {c}_{p}l{w}_{0}}{k}, J=\frac{kl}{\rho {c}_{p}{w}_{0}}, \Omega =\frac{\Omega {l}^{2}}{\nu }$$

$u$ and $w$ refer the velocity of fluid in $y$ and z direction respectively.${w}_{0}$ is represented as characteristic velocity. Fluid density is referred as $\rho$. Also, $\theta$ and $\phi$ refers as temperature and diffusivity of fluid. Da and $\Omega$ expressed as darcy number and rotational parameter, ${G}_{pm}\ and\ Pe$ referred as particle mass parameter (resistance parameter) and Peclet number in mass and heat transfer. Soret number is represented as $Sr$, heat conduction parameter is represented as $J$, ${T}_{c} \ and \ {C}_{c}$ referred as boundary temperature and concentration of CSF. ${T}_{0} \ and\ {C}_{0}$ refers free steam temperature and concentration of CSF. The following were the governing Eq. (1) to (5) in dimensionless form.

7 $$Re\left(\frac{\partial u}{\partial t}+G\frac{\partial u}{\partial y}\right)+2\Omega w=\frac{{\partial }^{2}u}{{\partial y}^{2}}+\frac{1}{Da}u-{G}_{pm}u$$

8 $$Re \left(\frac{\partial w}{\partial t}+G\frac{\partial w}{\partial y}\right)-2\Omega w=\frac{{\partial }^{2}w}{{\partial y}^{2}}+\frac{1}{Da}w-{G}_{pm}w$$

9 $$\frac{\partial \theta }{\partial t}+\frac{\partial \theta }{\partial y}=\frac{1}{Pe}\frac{{\partial }^{2}\theta }{{\partial y}^{2}}+J\theta$$

10 $$\frac{\partial \phi }{\partial t}+\frac{\partial \phi }{\partial y}=\frac{1}{Re Sc}\frac{{\partial }^{2}\phi }{{\partial y}^{2}}+\frac{Sr}{Re}\frac{{\partial }^{2}\theta }{{\partial y}^{2}}$$

corresponding initial and boundary conditions are,

$$u=0,w\,=\,0,\theta =\phi =1 \,at \,y\,=\,0$$

$$u=1,w\,=\,0,\theta =\phi =0 \,at\, y\,=\,1$$

$u$ and $w$ is inspected as a function of y since the fluid flows rotationally with respect to y axis.

To solve the above momentum equation, we introduce the complex velocity, $\mathrm{F }= u +iw$

The momentum equation in an explicit form of complex form is

11 $$Re\left(\frac{\partial F}{\partial t}+G\frac{\partial F}{\partial y}\right)+2\Omega IF=-\frac{2}{\rho }\frac{\partial p}{\partial x}+\frac{{\partial }^{2}F}{{\partial y}^{2}}-\frac{1}{Da}F-{G}_{pm}F$$

We assume a suitable boundary condition $F=0 , y=0,t\le 0$

$$F=1+ \frac{\varepsilon }{2}({e }^{i\lambda t} +{e }^{-i\lambda t}) , y=1,t>1$$

## Method of solution

We solve the above governing equation using the perturbation technique as the method is quite an error-less, assuming the trail solution for velocity, diffusivity, and heat transfer as

12 $$u\left( y, t\right) ={ u}_{0} \left(y\right)+\frac{\epsilon }{2}{e}^{i\lambda t}{u}_{1}\left(y\right) +\frac{\epsilon }{2}{e}^{-i\lambda t} {u}_{2}\left(y\right)$$

13 $$\theta ( y, t) ={ \theta }_{0} (y) +\frac{\epsilon }{2}{e}^{i\lambda t}{\theta }_{1} (y) +\frac{\epsilon }{2}{e}^{-i\lambda t} {\theta }_{2} (y)$$

14 $$\varphi ( y, t) ={ \varphi }_{0} (y) +\frac{\epsilon }{2}{e}^{i\lambda t}{\varphi }_{1} (y) +\frac{\epsilon }{2}{e}^{-i\lambda t} {\varphi }_{2} (y)$$

$\lambda$ refers to oscillation frequency and $\epsilon$ is an arbitrary constant parameter defined in such a way that$\epsilon \ll 1$. Let us consider ${u}_{0},{ u}_{1,}{ u}_{2}$, ${\theta }_{0}, { \theta }_{1,}{ \theta }_{2,} { \varphi }_{0},{ \varphi }_{1},{ \varphi }_{2}$ refers base part, first and second orders of momentum equation, energy equation, and concentration equation respectively.

Zero ($${F}_{0})$$, First $${(F}_{1})$$, Second ($${F}_{2})$$ order of complex form of the equation of motion:

15 $$\frac{1}{Re}\left(\frac{{\partial }^{2}{ F}_{0}}{{\partial y}^{2}}\right)- \frac{\partial {F}_{0}}{\partial y}-\frac{{R}_{m}}{Re}{ F}_{0}=0$$

16 $$\left(\frac{{\partial }^{2}{ F}_{1}}{{\partial y}^{2}}\right)-Re \frac{\partial {F}_{1}}{\partial y}-(Re+{R}_{m}){F}_{1}=0$$

17 $$\left(\frac{{\partial }^{2}{ F}_{2}}{{\partial y}^{2}}\right)-Re \frac{\partial {F}_{2}}{\partial y}-(Re+{R}_{m}){F}_{2}=0$$

were ${R}_{m}=$ $\frac{1}{Da}-{G}_{pm}-2\Omega I$ boundary conditions are,

$${F}_{0}{= F}_{1}={ F}_{2}=0 \,at \,y=0$$

$${F}_{0}{= F}_{1}={ F}_{2}=1\, at \, y=1$$

Zero (${\theta }_{0})$, First (${\theta }_{1})$, Second (${\theta }_{2})$ order of energy equations:

18 $$\left(\frac{{\partial }^{2}{ \theta }_{0}}{{\partial y}^{2}}\right)-Pe \frac{\partial {\theta }_{0}}{\partial y}+J Pe \ { \theta }_{0}=0$$

19 $$\left(\frac{{\partial }^{2}{ \theta }_{1}}{{\partial y}^{2}}\right)-Pe \frac{\partial {\theta }_{1}}{\partial y}+(J+i\lambda )Pe \ { \theta }_{1}=0$$

20 $$\left(\frac{{\partial }^{2}{ \theta }_{2}}{{\partial y}^{2}}\right)-Pe \frac{\partial {\theta }_{2}}{\partial y}+(J-i\lambda )Pe \ { \theta }_{2}=0$$

Then the corresponding boundary conditions are,

$${\theta }_{0}{= \theta }_{1}={ \theta }_{2}=1 \,at\, y=0$$

$${\theta }_{0}{= \theta }_{1}={ \theta }_{2}=0 \,at\, y=1$$

Zero (${\varphi }_{0})$, First (${\varphi }_{1})$, Second (${\varphi }_{2})$ order of concentration equation

21 $$\left(\frac{{\partial }^{2}{ \varphi }_{0}}{{\partial y}^{2}}\right)-Re \,Sc\frac{\partial {\varphi }_{0}}{\partial y}=-Sr \,Sc\left(\frac{{\partial }^{2}{ \theta }_{0}}{{\partial y}^{2}}\right)$$

22 $$\left(\frac{{\partial }^{2}{ \varphi }_{1}}{{\partial y}^{2}}\right)-Re \,Sc\frac{\partial {\varphi }_{1}}{\partial y}-(i\lambda\, Sc\, Re){\varphi }_{1}=-Sr\, Sc\left(\frac{{\partial }^{2}{ \theta }_{1}}{{\partial y}^{2}}\right)$$

23 $$\left(\frac{{\partial }^{2}{ \varphi }_{2}}{{\partial y}^{2}}\right)-Re \,Sc\frac{\partial {\varphi }_{2}}{\partial y}+\left(i\lambda\, Sc\, Re\right){\varphi }_{2}=-Sr \,Sc\left(\frac{{\partial }^{2}{ \theta }_{2}}{{\partial y}^{2}}\right)$$

$i$ represents the complex form of variable ${{\theta }_{1}{ ,\theta }_{2} , \varphi }_{1 },{ \varphi }_{2}$ of the above equations respectively.

Hence corresponding boundary conditions are,

$${\varphi }_{0}{= \phi }_{1}={ \phi }_{2}=1 \,at\, y=0$$

$${\varphi }_{0}{= \phi }_{1}={ \phi }_{2}=0 \,at\, y=1$$

The above equation is solved by the analytical way of perturbation method (Additional file 1: Appendix), hence, resultant equations can be expressed as

24 $${F}_{0}={A}_{1}{e}^{{m}_{1}y}+{A}_{2}{e}^{{m}_{2}y}$$

25 $${F}_{1}={A}_{3}{e}^{{m}_{3}y}+{A}_{4}{e}^{{m}_{4}y}$$

26 $${F}_{2}={A}_{5}{e}^{{m}_{5}y}+{A}_{6}{e}^{{m}_{6}y}$$

27 $${\theta }_{0}={A}_{7}{e}^{{m}_{7}y}+{A}_{8}{e}^{{m}_{8}y}$$

28 $${\theta }_{1}={A}_{9}{e}^{{m}_{9}y}+{A}_{10}{e}^{{m}_{10}y}$$

29 $${\theta }_{2}={A}_{11}{e}^{{m}_{11}y}+{A}_{12}{e}^{{m}_{12}y}$$

30 $${\phi }_{0}={A}_{13}{e}^{{m}_{13}y}+{A}_{14}{e}^{{m}_{14}y }+{A}_{19}{e}^{{m}_{7}y }+{A}_{20}{e}^{{m}_{8}y}$$

31 $${\phi }_{1}={A}_{15}{e}^{{m}_{15}y}+{A}_{16}{e}^{{m}_{16}y }+{A}_{21}{e}^{{m}_{9}y }+{A}_{22}{e}^{{m}_{10}y}$$

32 $${\phi }_{2}={A}_{17}{e}^{{m}_{17}y}+{A}_{18}{e}^{{m}_{18}y }+{A}_{23}{e}^{{m}_{11}y }+{A}_{24}{e}^{{m}_{12}y}$$

## Result and discussion

**Fig. 2:**
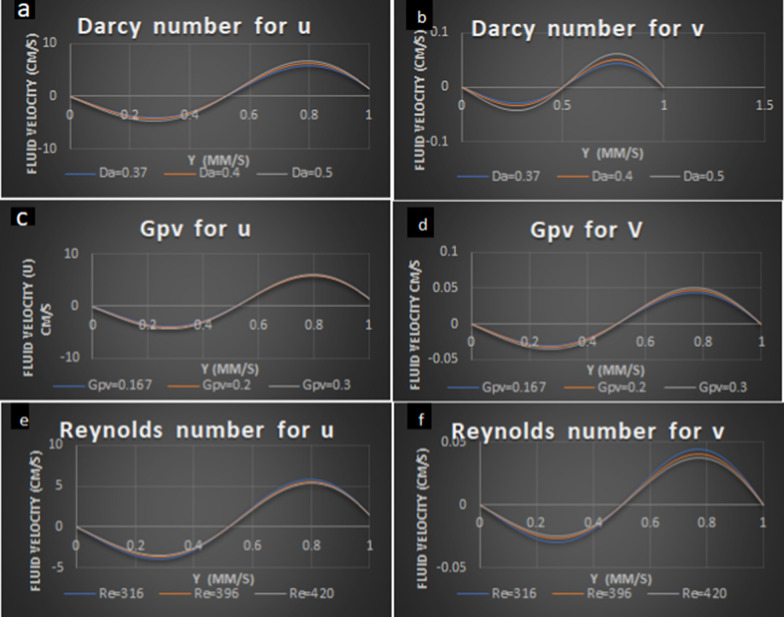
Analysis of CSF fluid in case of real and imaginary velocity with varying parameters

**Fig. 3:**
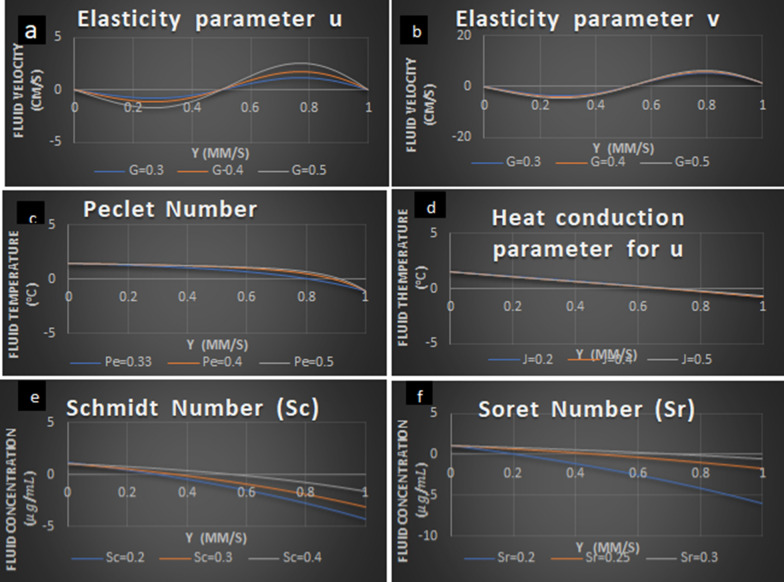
Analysis of fluid velocity, temperature, and diffusivity by varying parameters

In the present study, we adopt some default parameters using Additional file 1: Table 1. There are no relevant literature relating a few parameters like Soret number, and heat conduction parameter to compare with the following finding of our research article. In the following graphs, we summarized the system of governing Equations in the region, with the boundary conditions are solved analytically. To understand the behaviour of the oscillating flow characteristics, velocity $(F)$, temperature $\left(\theta \right)$ and Concentration $(\varphi )$ are calculated by varying the emerging flow parameters like Reynolds number, Peclet number, Darcy number, Soret number, Schmidt number, particle mass parameter, heat conduction parameter, etc. The velocity of CSF for patients varies from -15 m/s to 15 m/s for various parameters like Reynolds number, Darcy Number, Elasticity parameters etc.,

$t=0.01$, $\rho =998.2$, $$\begin {aligned}\lambda  =0.3, \epsilon =0.01, J = 0.4, \vartheta =0.8, k=0.67 \times {10}^{-16} ,\vartheta = 0.8,{G}_{pv}=0.167, \\  k=0.67\times {10}^{-16}, 150\le Re \le 420, Sr=0.2,Da=0.37 \end{aligned}$$ were the values assigned for the graphical representation of various parameters (Figs. 2 and 3).

The permeability of the fluid increased as there is a feasible signal in filaments present in the epidermal cells. Hence, Darcy's number increases as the velocity of the fluid increases gradually when the velocity of the fluid increases. This reflects that there is an increase in fluid in the porous medium say pia mater as the pressure increases. (Fig. 2a, b)

The resistance (particle mass parameter) increases at a peak level as the velocity increase, as the drop in fluid flow the resistance in the flow regions will be less (Fig. 2c, d)

The Reynolds number (Re) is used to predict the flow patterns for different fluids. It is the ratio of inertial forces to viscous forces within a fluid which is subjected to relative internal movement due to different fluid velocities. The inertial force increases predominantly to the viscosity of the fluid flow. It reflects that the hydrocephalus CSF fluid flow increases in SSS as there are drop-out fluid molecules in the third ventricle (Fig. 2e, f)

Elastic deformation plays a major role in CSF circulation. When the elasticity increases due to the bulk flow of hydrocephalus, for a certain time period as the flow is in a closed medium (Fig. 3a, b). As a result, this closed medium produces more enamor pressure than the normal subject when compared with hydrocephalic patients.

Peclet number is the measure of advective transport of fluid particles to mass diffusion rate. Here there is a decrease in the concentration of the fluid along its length due to the increase of Peclet number. Hence there is a transfer of heat when the diffusivity conduction with convection. As there is an excess secretion of CSF due to weak fluid particle movement which results in increases in the fluid temperature due to an increase in fluid velocity (Fig. 3c).

The heat conduction parameter is also increasing significantly showing that there is a decrease in thermal heat transfer when the fluid is in excess (Fig. 3d).

Schmidt number (Sc) is a change of momentum diffusivity with mass diffusivity and is used to characterize fluid flows with simultaneous momentum and mass diffusion convection processes. Here, the transport diffusivity decreases predominantly as the mass diffusivity decreases. As a result, the validity fluid shows that CSF concentration decreases as the Schmidt number increases (Fig. 3e) Soret number is the rate of change in temperature difference to the fluid concentration. In Figure 3f, an increase in Soret number shows larger temperature variations. Soret number plays a major role in this paper as there is temperature variation in the subarachnoid space when there is an increase in thermodynamical level enormously then it should be noted by the neurologist.

## Conclusion

The following were the conclusions that are made from the present investigation. The velocity of a fluid flow increases for increasing Darcy number, resistance parameter and Reynolds number. An increase in Elasticity with respect to dimensional change results in an increase in CSF velocity. There is a significant difference in temperature fall due to the enhancement of heat conduction parameter and Peclet number. The impact of the increase in Schmidt number, and Soret number considerably reduces the CSF fluid concentration. An increase in fluid velocity reflects significant changes in high intracranial pressure in the flow regime. It is observed that the comparison of the results produced using the revised elastic modulus with those of an existing value used in similar to simulations generated in [2, 4, 28]. Future works may generalize the present approach to consider a three-dimensional computational CSF pulsatile flow model for Non-Newtonian characteristics with pressure differences in the viscoelastic nature. There are a few limitations in this model, that is results were made to analyse CSF using the mathematical model with few parameters, but according to neurologists there a certain other parameters used in the three-dimensional model that have been simulated in various perpontine region in brain which is quite smaller visible only through CINE-MRI scan. We attribute this criterial result in the future to include more pulsatility model that the outcome is more appropriate one.

### Supplementary Information

**Additional file 1:** **Appendix.** Obtained from solving the governing equations Eqns. (6)-(9) from manuscript. **Table S1.** Properties involved in CSF flow used to validate various parameters.

## Data Availability

All data used for the present study are available and could be requested from the authors.

## Abbreviations

CSF: Cerebrospinal fluid
u,w: Velocity of CSF flow in y and z direction
CNS: Cental nervous system
ICP: Intracranial pressure
SSS: Subarachnoid space
ρ: Fluid density
ν: Kinematic viscosity
K: Permeabilty of porous layer (pia mater)
w_0_: Characteristic velocity
σ: Electric conductivity
F: Velocity of real and imaginary part
I: Imaginary part
R: Resistance parameter (Stokes resistance)
c_p_: Specific heat capaity at Constant pressure
T_c_: Wall temperature of CSF flow
C_c_: Wall concentration of CSF flow
T_0_: Steam temperature of CSF flow
C_0_: Steam concentration of CSF flow
Ω′: Rotation parameter
Ω: Dimensionless rotation parameter
Re: Reynolds number
J: Heat conduction parameter
ε: Pertubation parameter ε ≪ 0
λ: Pertubation postive real constant
k: Thermal conductivity
K_T_: Thermal diffusion ratio
N: Number density
t: Dimensionless time taken
Da: Darcy number
G_pm_: Particle mass parameter
Pe: Peclet number
Sr: Soret number
Sc: Schmidt number for mass transfer
θ: Temperature of fluid flow in brain
φ: Transport diffusivity of the fluid
y and z: Co-ordinate system
