# Supplementary material for: Impact of thermodynamical rotational flow of cerebrospinal fluid in the presence of elasticity
Source: BMC Res Notes. 2023 Nov 29;16:355. doi: 10.1186/s13104-023-06602-w (PMC10688068; doi:10.1186/s13104-023-06602-w)
Supplement: Supplementary file 1 — Additional file 1: Appendix. Obtained from solving the governing equations Eqns. (6)-(9) from manuscript. Table S1. Properties involved in CSF flow used to validate various parameters. [file 13104_2023_6602_MOESM1_ESM.docx]

**Impact of Thermodynamical Rotational Flow of Cerebrospinal Fluid in the Presence of Elasticity**

^*^Hemalatha Balasundaram

**^*^Corresponding author:** Hemalatha Balasundaram, Ph.D.,

Department of Mathematics,

Rajalakshmi Institute of Technology,

Chembarambakkam, Chennai,

Tamil Nadu 600124, India.

Phone: +91 (978)69 01291

E-mail: hemalatha.b@ritchennai.edu.in

ORCID ID: 0000-0001-7451-693X

**Additional material 1.**

**Appendix:** Obtained from solving the governing equations Eqns. (6)-(9) from manuscript.

**Table S1:** Properties involved in CSF flow used to validate various parameters

**Appendix:**

$$m_{1}=\frac{Re+\sqrt{{Re}^{2}+4R}}{2}{; m}_{2}=\frac{Re-\sqrt{{Re}^{2}+4R}}{2}$$

$$m_{3}=\frac{Re+\sqrt{{Re}^{2}+4(R+Re)}}{2}{; m}_{4}=\frac{Re-\sqrt{{Re}^{2}+4(R+Re)}}{2}$$

$$m_{5}=\frac{Re+\sqrt{{Re}^{2}+4(R+Re)}}{2}{; m}_{6}=\frac{Re-\sqrt{{Re}^{2}+4R(R+Re)}}{2}$$

$$R=\frac{1}{Da}-Gpm-2\Omega I$$

$A_{1}+A_{2}=0$

$A_{3}+A_{4}=0$

$$A_{5}+A_{6}=0$$

$A_{2}=\frac{1}{e^{m_{2}}-e^{m_{1}}}$ , $A_{4}=\frac{1}{e^{m_{4}}-e^{m_{3}}}$ , $A_{6}=\frac{1}{e^{m_{6}}-e^{m_{5}}}$

$$m_{7}=\frac{Pe+\sqrt{{Pe}^{2}+4JPe}}{2}{; m}_{8}=\frac{Pe-\sqrt{{Pe}^{2}+4JPe}}{2}$$

$$m_{9}=\frac{Pe+\sqrt{{Pe}^{2}+4JPe}}{2}{; m}_{10}=\frac{Pe-\sqrt{{Pe}^{2}+4JPe}}{2}$$

$$m_{11}=\frac{Pe+\sqrt{{Pe}^{2}+4(J+i\lambda)Pe}}{2}{; m}_{12}=\frac{Pe-\sqrt{{Pe}^{2}+4(J+i\lambda)Pe}}{2}$$

$$m_{13}=\frac{Pe+\sqrt{{Pe}^{2}+4(J-i\lambda)Pe}}{2}{; m}_{14}=\frac{Pe-\sqrt{{Pe}^{2}+4(J-i\lambda)Pe}}{2}$$

$A_{7}=1-A_{8}$, $A_{9}=1-A_{10}$,$A_{11}=1-A_{12}$

$A_{8}=\frac{e^{m_{7}}}{e^{m_{7}}-e^{m_{8}}}$ , $A_{10}=\frac{e^{m_{9}}}{e^{m_{9}}-e^{m_{10}}}$ , $A_{12}=\frac{e^{m_{11}}}{e^{m_{11}}-e^{m_{12}}}$

$$\frac{\partial^{2}\theta_{0}}{\partial y^{2}}=A_{7}e^{m_{1}y}+A_{8}e^{m_{2}y}$$

$$\frac{\partial^{2}\theta_{1}}{\partial y^{2}}=A_{9}e^{m_{3}y}+A_{10}e^{m_{4}y}$$

$$\frac{\partial^{2}\theta_{2}}{\partial y^{2}}=A_{11}e^{m_{5}y}+A_{12}e^{m_{6}y}$$

$A_{7}={m_{1}}^{2}A_{1}$, $A_{8}={m_{2}}^{2}A_{2}$

$A_{9}={m_{3}}^{2}A_{3}$, $A_{10}={m_{4}}^{2}A_{4}$

$A_{11}={m_{5}}^{2}A_{5}$, $A_{12}={m_{6}}^{2}A_{6}$

$A_{19}=\frac{Sr A_{7}}{Re m_{1}(m_{1}-Sc Re)}$ , $A_{20}=\frac{Sr A_{8}}{Re m_{2}(m_{2}-Sc Re)}$

$A_{21}=\frac{Sr A_{11}}{Re ({m_{3}}^{2}- m_{3} Sc Re+Sc Re i\lambda)}$ , $A_{22}=\frac{Sr A_{12}}{Re ({m_{4}}^{2}- m_{4} Sc Re+Sc Re i\lambda)}$

$A_{23}=\frac{Sr A_{11}}{Re ({m_{5}}^{2}- m_{5} Sc Re+Sc Re i\lambda)}$ , $A_{24}=\frac{Sr A_{12}}{Re ({m_{6}}^{2}- m_{6} Sc Re+Sc Re i\lambda)}$

$m_{6}=Sc Re,$

$m_{13}=\frac{Sc Re+\sqrt{{(Sc Re)}^{2}+4Sc Re i\lambda)}}{2}$ , $m_{14}=\frac{Sc Re-\sqrt{{(Sc Re)}^{2}+4Sc Re i\lambda)}}{2}$

$m_{15}=\frac{Sc Re+\sqrt{{(Sc Re)}^{2}-4Sc Re i\lambda)}}{2}$ , $m_{16}=\frac{Sc Re-\sqrt{{(Sc Re)}^{2}-4Sc Re i\lambda)}}{2}$

$$A_{14}=\frac{1}{1-e^{m_{6}}}\left[ A_{19}\left( 1-e^{m_{1}} \right)+A_{20}\left( 1-e^{m_{2}} \right)-1 \right]$$

$$A_{13}=A_{19}+A_{20}-A_{14}$$

$$A_{15}=A_{21}+A_{22}-A_{16}$$

$$A_{16}=\frac{1}{e^{m_{7}}-e^{m_{8}}}\left[ A_{21}\left( 1-e^{m_{3}} \right)+A_{22}\left( 1-e^{m_{4}} \right)-1 \right]$$

$$A_{18}=A_{23}+A_{24}-A_{17}$$

$$A_{17}=\frac{1}{e^{m_{9}}-e^{m_{10}}}\left[ A_{23}\left( e^{m_{9}}-e^{m_{5}} \right)+A_{24}\left( e^{m_{9}}-e^{m_{6}} \right)-1 \right]$$

**Table S1.** Properties involved in CSF flow used validate various parameters

| Physical constant | Value |
| --- | --- |
| Density | $998.2$ [17] |
| Dynamic viscosity $Ns/m^{2}$ | $8.91\times{10}^{-4}$ [12] |
| Elasticity $N/m^{2}$ | $1\times{10}^{4}$ [12] |
| Reynolds number | $150-420$ [9] |
| Darcy number | 0.37 [9] |
| Skin Temperature | $2-4℃$and more [6] |
| Thermal conductivity $(w/m/K)$ | 0.63 [15] |
| Thermal diffusivity ($m^{2}/s)$ | $1.4\times{10}^{-7}$ [15] |
| Specific heat | 4.19 [2] |
| Heat conduction parameter | 0.4 |
| Schmidt Number (water) | 0.2 |
| Resistance parameter Gpv | 0.167 [18] |
| Thermal resistance | $2.2\times{10}^{4}$ [2] |
| Soret Number | 0.2 |
